# Prediction of functional outcome using the novel asymmetric middle cerebral artery index in cryptogenic stroke patients

**DOI:** 10.1371/journal.pone.0208918

**Published:** 2019-01-02

**Authors:** Minho Han, Young Dae Kim, Hyung Jong Park, In Gun Hwang, Junghye Choi, Jimin Ha, Ji Hoe Heo, Hyo Suk Nam

**Affiliations:** 1 Department of Neurology, Yonsei University College of Medicine, Seoul, Korea; 2 Department of Science for Aging, Yonsei University Graduate School, Seoul, Korea; 3 Brain Korea 21 Plus Project for Medical Science, Yonsei University, Seoul, Korea; Universitatsklinikum Freiburg, GERMANY

## Abstract

**Background:**

Etiology is unknown in approximately one-quarter of stroke patients after evaluation, which is termed cryptogenic stroke (CS). The prognosis of CS patients is largely undetermined. We created a novel index from transcranial Doppler parameters including mean flow velocity (MV) and pulsatility index (PI) and investigated whether the calculation of asymmetry in the novel parameter can predict functional outcomes in CS patients.

**Methods:**

We made the middle cerebral artery (MCA) index (%) as a novel parameter, which was calculated as 100 X (MCA MV + MCA PI X 10) / (MCA MV–MCA PI X 10). The MCA asymmetry index (%) was also calculated as 100 X (|Rt MCA index–Lt MCA index|) / (Rt MCA index + Lt MCA index) / 2. Poor functional outcomes were defined as modified Rankin Scale score (mRS) ≥3 at 3 months after stroke onset.

**Results:**

A total of 377 CS patients were included. Among them, 52 (13.8%) patients had a poor outcome. The overall MCA asymmetry index was two-fold higher in CS patients with a poor outcome (10.26%) compared to those with a good outcome (5.41%, p = 0.002). In multivariable analysis, the overall MCA asymmetry index (OR, 1.054, 95% CI, 1.013–1.096, p = 0.009) and the cutoff value of the overall MCA asymmetry index >9 were associated with poor outcomes at 3 months (OR, 3.737, 95% CI, 1.530–9.128, p = 0.004).

**Conclusion:**

We demonstrated that the novel asymmetric MCA index can predict short-term functional outcomes in CS patients.

## Introduction

Etiology is unknown in approximately 20% to 25% of stroke patients despite extensive evaluation, which is termed cryptogenic stroke (CS). The occurrence of CS is more frequent in young stroke patients than in older patients. To be classified as CS, neither significant cerebral artery stenosis nor cardioembolic sources was found [1]. Several studies reported that 9% to 30% of CS patients suffer recurrent stroke [2], and 23% to 35% have poor short-term outcomes [3].

Transcranial Doppler (TCD) can evaluate basal cerebral arteries with hemodynamic information, including mean flow velocity (MV) and pulsatility index (PI). Abnormal hemodynamics and cerebral artery stenosis can also be non-invasively identified using TCD. TCD parameters are influenced and changed by various factors, including infarct size and asymmetric lesions [4, 5]. Therefore, the evaluation of asymmetry using TCD might be beneficial for the prediction of outcomes.

Previous studies demonstrated that unilateral TCD parameters can predict prognosis in patients with large artery atherosclerosis or small artery disease [6, 7]. However, to the best of our knowledge, no study has yet investigated the association between TCD parameter asymmetry and the prognosis of CS patients. In this regard, we made a novel index that augments the difference in bilateral TCD parameters. We determined whether the calculation of asymmetry in this novel parameter can predict functional outcomes at 3 months in CS patients.

## Materials and methods

### Patients and evaluation

We reviewed prospectively collected data from the Yonsei Stroke Registry. From January 2007 to June 2013, a consecutive 3738 patients with ischemic stroke or transient ischemic attack within 7 d were admitted and registered in a prospective stroke registry [8].

During admission, all patients were thoroughly evaluated for medical history, clinical manifestations, and vascular risk factors. Each patient was evaluated with 12-lead electrocardiography, chest x- ray, lipid profiles, and standard blood tests. All registered patients underwent brain imaging studies, including brain computed tomography (CT) and/or magnetic resonance imaging (MRI). Angiographic studies using CT angiography, magnetic resonance angiography, or digital subtraction angiography were included in standard evaluations. Neurosonographic evaluation, including TCD and carotid Doppler, were routinely performed. Additional blood tests for coagulopathy or prothrombotic conditions were conducted in patients younger than 45-years-old. Transesophageal echocardiography was included in the standard evaluation, except in patients with decreased consciousness, impending brain herniation, poor systemic condition, inability to accept an esophageal transducer due to swallowing difficulty or tracheal intubation, or lack of informed consent [9]. Transthoracic echocardiography, heart CT, and Holter monitoring were also performed in selected patients [10]. Most patients were admitted to the stroke unit and monitored continuously with EKG during their stays. This study was approved by the institutional review board of Severance Hospital, Yonsei University Health System.

### Stroke subtype classification

Stroke classifications were determined during weekly conferences based on a consensus of stroke neurologists. Data, including clinical information, risk factors, imaging study findings, laboratory analyses, and other special evaluations were collected. Along with these data, prognosis during hospitalization and long-term outcomes were also determined. Data were entered into a web-based registry. Stroke subtypes were identified according to the Trial of ORG 10172 in Acute Stroke Treatment (TOAST) classification [11]. We defined CS as strokes of undetermined etiology attributable to negative evaluation, despite extensive work-up.

### TCD parameters

Patients underwent TCD examination (Nicolet TC8080, Stockport, UK) within 7 d of admission. All TCD recordings were carried out by two medical technicians. In all patients, peak systolic flow velocity (PSV) and end-diastolic flow velocity (EDV) were measured with a handheld 2-MHz probe in the middle cerebral artery (MCA). The MCA was insonated through the trans-temporal window at various depths of 44–68 mm. The MV value was automatically calculated by a Doppler machine using the mean of five cycles (according to the formula MV = EDV + (PSV–EDV) / 3. The PI value was simultaneously calculated as (PSV–EDV) ⁄ MV. The recorded MV and PI were measured at a minimum of two depths to obtain hemodynamic information on the proximal MCA (M1, 58–68 mm) and the distal MCA (M2, 44-56mm) [12].

We developed the MCA index, which combines the effect of MV and PI of the MCA to overcome the limitation of single TCD parameters of MV or PI. The formula of the MCA index (%) is 100 X (MCA MV + MCA PI X 10) / (MCA MV–MCA PI X 10). Mathematically, the MCA index will become high when MV is low and PI is high. We performed a simulation to calculate the MCA index when the MV or the PI values are changed, and found that PI multiplied by 10 is most suitable to augment differences (described in S1 Table). The mean MCA index was calculated as (proximal MCA index + distal MCA index) / 2. According to previous reports, an asymmetry index of the MCA index, MV, and PI was calculated as 100 X (|Rt–Lt|) / (Rt + Lt) / 2 [13]. Overall MCA asymmetry index was calculated as 100 X (|Rt mean MCA index–Lt mean MCA index |) / (Rt mean MCA index + Lt mean MCA index) / 2. The intraclass correlation coefficients (ICCs) for TCD parameters were yielded to investigate the inter-rater reliability between investigators.

### Demographic characteristics and risk factors

We collected baseline characteristics for sex, age, risk factors, and initial neurological deficit (National Institutes of Health Stroke Scale score, NIHSS score) at admission. Hypertension was diagnosed in which a patient was on antihypertensive medication or had systolic arterial pressures ≥140 mm Hg or diastolic arterial pressures ≥90 mm Hg on repeated measurements during admission. Diabetes mellitus was diagnosed in which a patient had taken an oral hypoglycemic agent or insulin, or had fasting plasma glucose ≥7.0 mmol/L. Hypercholesterolemia was diagnosed in which a patient had taken lipid-lowering agents after diagnosis of hypercholesterolemia or had low-density lipoprotein cholesterol ≥4.1 mmol/L, or total cholesterol ≥6.2 mmol/L. A current smoker was defined as having smoked a cigarette within 1 year prior to admission. Peripheral artery disease was determined when the patient had an ankle-brachial index <0.9 [14].

### Follow-up and outcome measures

Stroke-related functional outcome was assessed using the modified Rankin Scale score (mRS) via a direct interview performed by a physician or through a telephone interview conducted by a well-trained research nurse after 3 months from stroke onset. The mRS consists of six different grades of disability, from 0 for “no symptoms at all,” to 5 for “severe disability or bedridden, incontinent, and requiring constant nursing care and attention,” to grade 6 for “death”. A poor functional outcome was defined as mRS ≥3 at 3 months after stroke onset [1].

### Standard protocol approvals, registrations, and patient consents

The institutional review board of Severance Hospital, Yonsei University Health System, approved this study and waived the patients' informed consent because of a retrospective design and observational nature of this study.

### Statistical analysis

SPSS for Windows (version 20, SPSS, Chicago, IL, U.S.A.) was used for statistical analysis.

The patients were divided into two groups according to the mRS at 3 months after stroke onset. The statistical significance of intergroup differences was assessed using the χ2 or Fisher’s exact tests for categorical variables, and independent two sample t test or Mann-Whitney U test for continuous variables. Data are expressed as means ± standard deviation or medians (interquartile ranges) for continuous variables and number (%) for categorical variables. The receiver operating characteristic (ROC) analysis was performed to identify the optimal cutoff value of the overall MCA asymmetry index with the highest Youden index (sensitivity + specificity– 1). We performed multivariable logistic regression with adjustments for confounding factors to investigate the association of the novel MCA asymmetry index with short-term functional outcomes in CS patients.

## Results

### Patient enrollments

**Fig 1:**
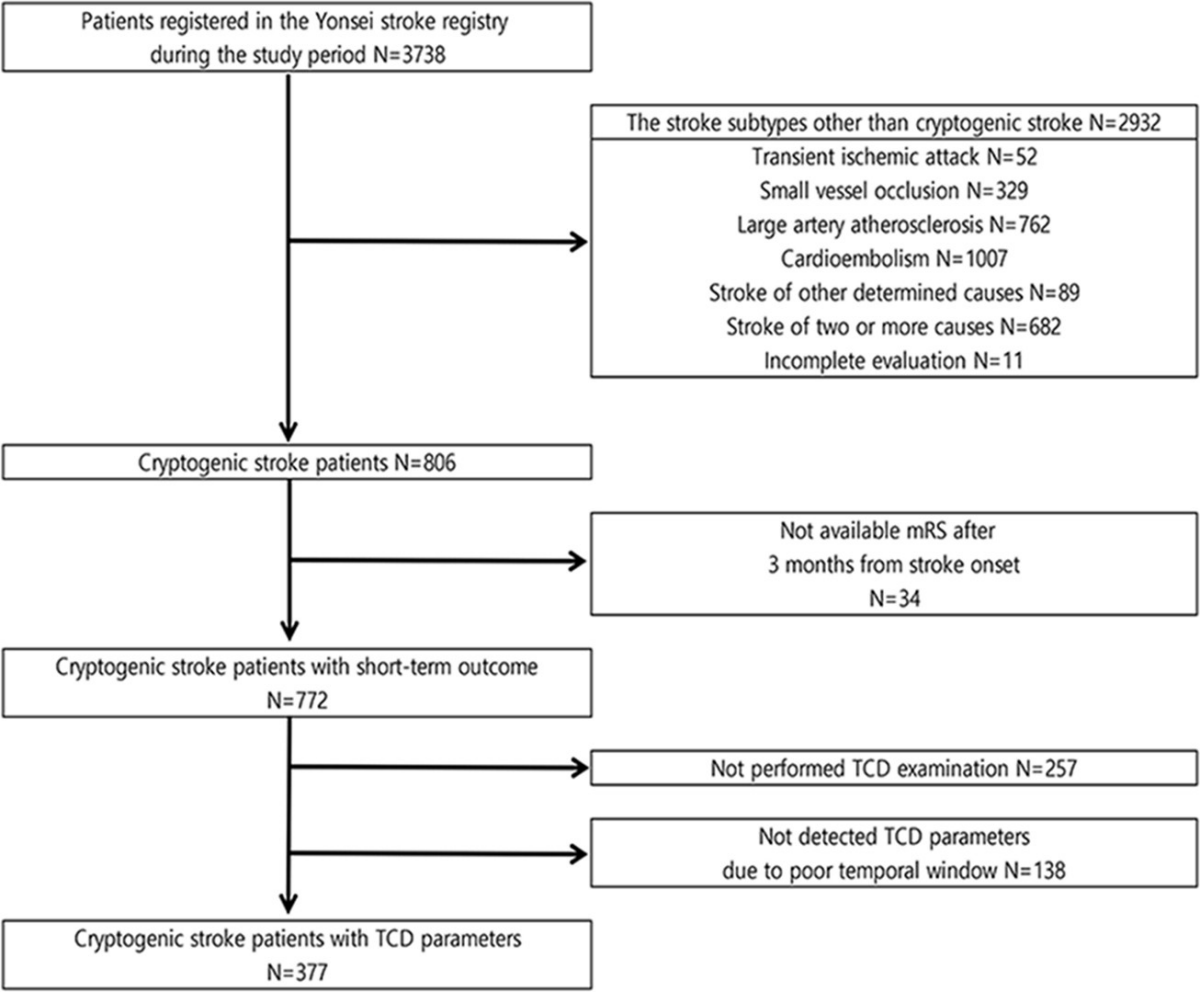
Flow chart of inclusion and exclusion criteria. The mRS indicates modified Rankin Scale.

During the study period, 3738 consecutive patients with acute ischemic stroke were registered to the Yonsei Stroke Registry. Exclusion criteria of this study were patients with stroke subtypes other than cryptogenic stroke, including transient ischemic attack (n = 52), small vessel occlusion (n = 329), large artery atherosclerosis (n = 762), cardioembolism (n = 1007), stroke of other determined causes (n = 89), stroke of two or more causes (n = 682), incomplete evaluation (n = 11), and follow-up loss (n = 34). In addition, patients who could not obtain TCD parameters (not undergoing TCD examination; n = 257, poor temporal window; n = 138) were excluded in the statistical analysis (Fig 1). After exclusion, a total of 377 CS patients were finally enrolled in this study.

### Demographic characteristics

Among the 377 CS patients, mean age was 62.10 ± 12.31 years and men were 275 (72.9%). 52 patients (13.8%) had a poor functional outcome. Univariable analysis revealed that poor functional outcomes at 3 months were associated with old age, men, initial stroke severity, longer time from admission to TCD, low level of hemoglobin, and high level of erythrocyte sedimentation rate, and D-dimer (Table 1, all p values <0.05).

**Table 1:** Demographic characteristics and comparison between patients with good outcomes and poor outcomes.

	Total	Good outcome (mRS 0–2; n = 325)	Poor outcome (mRS 3–6; n = 52)	p-value
	(n = 377)			
Age, y	62.10 ± 12.31	61.24 ± 12.19	67.46 ± 11.83	0.001
Men	275 (72.9)	243 (74.8)	32 (61.5)	0.046
NIHSS score at admission	2.0 [1.0, 4.0]	2.0 [1.0, 4.0]	8.0 [4.0, 15.0]	<0.001
Time from admission to TCD, day	3.0 [2.0, 4.0]	3.0 [2.0, 4.0]	4.0 [3.0, 6.0]	<0.001
Systolic blood pressure, mmHg	155.0 [136.0, 177.0]	156.0 [137.0, 179.0]	153.0 [132.0, 169.0]	0.109
Diastolic blood pressure, mmHg	87.0 [77.0, 96.0]	87.0 [78.0, 97.0]	85.0 [75.0, 90.0]	0.060
Thrombolysis therapy	18 (4.8)	14 (4.3)	4 (7.7)	0.291
**Risk factors**				
Hypertension	276 (73.2)	242 (74.5)	34 (65.4)	0.170
Diabetes mellitus	108 (28.6)	95 (29.2)	13 (25.0)	0.531
Hypercholesterolemia	62 (16.4)	51 (15.7)	11 (21.2)	0.323
Current smoker	123 (32.6)	110 (33.8)	13 (25.0)	0.206
Coronary artery disease	66 (16.4)	57 (17.5)	9 (17.3)	0.968
Peripheral artery disease	7 (1.9)	7 (2.2)	0 (0.0)	0.600
**Laboratory findings**				
Hemoglobin, g/dL	14.3 [13.1, 15.4]	14.5 [13.3, 15.5]	13.6 [11.7, 14.9]	0.001
ESR, mm/h	13.5 [7.0, 26.0]	13.0 [7.0, 26.0]	20.5 [8.0, 39.3]	0.014
D-dimer, μg/L	151.5 [77.8, 304.8]	129.0 [75.0, 271.3]	298.5 [127.8, 1206.8]	<0.001
Total cholesterol, mg/dL	178.0 [151.8, 202.3]	177.5 [151.8, 202.0]	178.0 [150.5, 210.3]	0.916
LDL, mg/dL	108.3 [86.8, 132.6]	108.4 [86.8, 132.6]	107.8 [87.5, 140.1]	0.493
Glucose, mg/dL	120.0 [104.0, 146.3]	120.0 [105.0, 147.3]	116.0 [101.0, 135.5]	0.177

Data are expressed as mean ± SD, median [interquartile rage], or a number (%)

mRS, modified Rankin Scale score; NIHSS, National Institutes of Health Stroke Scale; ESR, erythrocyte sedimentation rate; LDL, low density lipoprotein.

### TCD parameters

Among the classic TCD parameters (Table 2), patients with a poor outcome tended to have lower MV, and higher PI and asymmetry index. The asymmetry index of the distal MCA MV was higher in patients with a poor outcome (21.27%) compared to a good outcome (15.69%) (p = 0.049). Moreover, the overall asymmetry index of the MCA MV was higher in patients with a poor outcome (17.21%) compared to a good outcome (13.73%) (p = 0.051). The patients with a poor outcome exhibited higher distal PI on the right MCA (0.90 of a poor outcome vs. 0.84 of a good outcome, p = 0.043).

**Table 2:** Comparison of TCD parameters between patients with good outcomes (mRS 0–2) and poor outcomes (mRS 3–6) at 3 months.

	Poor outcome (n = 52)	Good outcome (n = 325)	p-value
**Mean flow velocity (MV) (cm/s)**			
** **Rt proximal MCA	52.5 [42.0, 63.3]	56.0 [46.0, 69.0]	0.067
** **Lt proximal MCA	55.0 [41.0, 70.0]	57.0 [47.0, 74.0]	0.313
** **Proximal MCA MV asymmetry index	18.54 [8.08, 34.01]	15.13 [7.14, 24.46]	0.170
Rt distal MCA	55.0 [40.3, 70.5]	54.0 [43.0, 69.0]	0.628
** **Lt distal MCA	57.0 [43.5, 69.0]	56.0 [46.0, 70.0]	0.898
** **Distal MCA MV asymmetry index	21.27 [11.20, 42.93]	15.69 [7.06, 29.92]	0.049
** **Rt mean MCA	53.5 [42.9, 65.1]	54.5 [45.0, 69.0]	0.217
** **Lt mean MCA	55.5 [42.3, 69.5]	57.0 [47.0, 72.0]	0.559
** **Overall MCA MV asymmetry index	17.21 [9.45, 42.89]	13.73 [6.23, 25.26]	0.051
**Pulsatility index (PI)**			
** **Rt proximal MCA	0.91 [0.77, 1.00]	0.87 [0.76, 1.00]	0.154
** **Lt proximal MCA	0.89 [0.76, 1.00]	0.85 [0.73, 1.00]	0.232
** **Proximal MCA PI asymmetry index	10.00 [6.94, 19.53]	9.23 [4.71, 16.09]	0.117
** **Rt distal MCA	0.90 [0.78, 1.10]	0.84 [0.73, 1.00]	0.043
** **Lt distal MCA	0.88 [0.62, 1.08]	0.84 [0.71, 1.00]	0.204
** **Distal MCA PI asymmetry index	10.62 [7.04, 27.25]	9.52 [4.20, 17.82]	0.087
** **Rt mean MCA	0.91 [0.79, 1.05]	0.86 [0.76, 0.98]	0.057
** **Lt mean MCA	0.92 [0.76, 0.99]	0.84 [0.73, 0.97]	0.155
** **Overall MCA PI asymmetry index	10.38 [4.21, 17.30]	8.56 [3.97, 13.74]	0.119
**Novel TCD parameters (%)**			
** **Rt proximal MCA index	141.14 [132.07, 162.77]	136.36 [126.42, 150.00]	0.046
** **Lt proximal MCA index	137.74 [126.58, 157.17]	134.33 [124.79, 148.89]	0.232
** **Proximal MCA asymmetry index	9.06 [2.52, 16.90]	5.88 [2.91, 10.55]	0.075
** **Rt distal MCA index	139.80 [128.97, 163.14]	136.84 [126.75, 152.50]	0.262
** **Lt distal MCA index	136.29 [130.30, 153.33]	135.56 [125.21, 149.29]	0.378
** **Distal MCA asymmetry index	10.38 [3.14, 22.05]	6.05 [3.00, 12.78]	0.046
** **Rt mean MCA index	140.12 [133.13, 156.97]	137.61 [127.54, 149.60]	0.117
** **Lt mean MCA index	137.27 [129.86, 154.67]	135.27 [125.99, 149.57]	0.308
** **Overall MCA asymmetry index	10.26 [3.77, 16.06]	5.41 [2.76, 10.42]	0.002

Data are expressed as mean ± SD, median [interquartile rage], or a number (%)

The MCA indices (%) are calculated as 100 X (MCA MV + MCA PI X 10) / (MCA MV–MCA PI X 10); The mean MCA index is calculated as (proximal MCA index + distal MCA index) / 2

An asymmetry index of the MCA index, MV, and PI was calculated as 100 X (|Rt–Lt|) / (Rt + Lt) / 2

mRS, modified Rankin Scale score; Rt, right; Lt, left; MCA, middle cerebral artery.

For the novel MCA index, patients with a poor outcome had a higher MCA index compared to those with a good outcome, and especially the right proximal MCA index was significantly different (141.14% of a poor outcome vs. 136.36% of a good outcome, p = 0.046). The asymmetry index of the distal MCA index was higher in patients with a poor outcome (10.38%) compared to a good outcome (6.05%) (p = 0.046). In addition, the overall asymmetry index of the MCA index was two-fold higher in patients with a poor outcome (10.26%) compared to a good outcome (5.41%) (p = 0.002). Inter-rater reliability between investigators for TCD parameters was fair to good as follows; rt M1 MV (ICC = 0.959, p<0.001), rt M2 MV (ICC = 0.947, p<0.001), lt M1 MV (ICC = 0.953, p<0.001), lt M2 MV (ICC = 0.974, p<0.001), rt M1 PI (ICC = 0.703, p = 0.042), rt M2 PI (ICC = 0.773, p = 0.019), lt M1 PI (ICC = 0. 594, p = 0.098), lt M2 PI (ICC = 0. 877, p = 0.002).

### Multivariable analysis

We performed a multivariable analysis after adjusting sex, age, and variables that exhibited a p value <0.05 in the univariable analyses (Table 3). The MCA asymmetry indices still showed an independent association with poor outcomes; the right proximal MCA index (odds ratio [OR], 1.018, 95% confidence interval [CI], 1.006–1.030, p = 0.003), the proximal MCA asymmetry index (OR, 1.040, 95% CI, 1.003–1.078, p = 0.032), and the overall MCA asymmetry index (OR, 1.054, 95% CI, 1.013–1.096, p = 0.009), respectively. The cutoff value of the overall MCA asymmetry index from the ROC curve analysis was >9, which was a strong independent predictor (OR, 3.737, 95% CI, 1.530–9.128, p = 0.004) (described in S1 Fig). Even if a poor functional outcome was defined more conservatively by mRS ≥2, the results were similar (described in S5 Table).

**Table 3:** Predictors of poor functional outcomes at 3 months.

	Univariable		Multivariable*	
	OR (95% CI)	p-value	OR (95% CI)	p-value
Men	0.540 (0.293–0.996)	0.048		
Age, y	1.048 (1.019–1.077)	0.001		
NIHSS score at admission	1.285 (1.198–1.378)	<0.001		
Hemoglobin, g/dL	0.796 (0.696–0.910)	0.001		
ESR, mm/h	1.017 (1.005–1.029)	0.007		
D-dimer, μg/L	1.000 (1.000–1.000)	0.002		
Time from admission to TCD, day	1.193 (1.072–1.328)	0.001		
**Novel TCD parameters**				
Rt proximal MCA index	1.012 (1.003–1.020)	0.009	1.018 (1.006–1.030)	0.003
Lt proximal MCA index	1.008 (0.997–1.018)	0.140	1.012 (0.998–1.025)	0.096
Proximal MCA asymmetry index	1.038 (1.007–1.069)	0.014	1.040 (1.003–1.078)	0.032
Rt distal MCA index	1.005 (0.999–1.010)	0.105	1.003 (0.996–1.010)	0.425
Lt distal MCA index	1.007 (1.000–1.014)	0.057	1.008 (0.999–1.017)	0.073
Distal MCA asymmetry index	1.027 (1.009–1.044)	0.002	1.020 (0.999–1.042)	0.061
Rt mean MCA index	1.008 (0.999–1.017)	0.066	1.008 (0.996–1.019)	0.212
Lt mean MCA index	1.011 (1.001–1.020)	0.025	1.014 (1.001–1.026)	0.034
Overall MCA asymmetry index	1.055 (1.026–1.086)	<0.001	1.054 (1.013–1.096)	0.009
Cutoff value of overall MCA asymmetry index >9	3.528 (1.897–6.561)	<0.001	3.737 (1.530–9.128)	0.004

Data were derived from logistic regression analysis

NIHSS, National Institutes of Health Stroke Scale; ESR, erythrocyte sedimentation rate; Rt, right; Lt, left; MCA, middle cerebral artery; OR, odds ratio; CI, confidence interval.

* adjusted for sex, age, NIHSS score at admission, hemoglobin, ESR, D-dimer, and Time from admission to TCD.

## Discussion

We demonstrated that a higher MCA asymmetry index was associated with poor functional outcomes at 3 months in CS patients. Adoption of TCD in the evaluation of CS patients provides not only unilateral cerebral hemodynamics, but also the novel MCA index and the MCA asymmetry index, which reflect bilateral hemodynamic differences. In this study, we found that the latter was a predictor of functional outcomes in CS patients.

The factors related to poor outcomes in CS patients are unclear. Concealed accompanying burdens of less than 50% atherosclerosis, cardioembolic sources like paroxysmal atrial fibrillation, or accompanying small artery diseases including white matter hyperintensity, can affect the outcomes of CS patients [1]. Moreover, hemodynamic impairment, changes in cerebral autoregulation, and vasospasm constitute other possible prognostic factors [15].

Abnormal hemodynamics and cerebral artery stenosis can be non-invasively determined by TCD. TCD parameters may sensitively reflect the changes of cerebral blood flow because TCD can evaluate the hemodynamics of cerebral blood flow in real time. In addition, by comparing the bilateral temporal window, TCD can provide the asymmetric distribution of cerebral blood flow. Among TCD parameters, MV reflects a relative integrity of cerebral arterial perfusion, whereas PI is associated with cerebrovascular resistance or intracranial compliance. Many TCD-based studies in stroke patients have been conducted using MV and PI. Increased MV was closely related to severe stenosis of relevant intracranial cerebral arteries, and increased PI was associated with small artery disease in diabetes patients and a larger infarction in lacunar stroke [6, 16, 17]. In addition, PI could play a role in estimating intracranial pressure [18].

Several studies were conducted to elucidate the association between TCD parameters and prognosis of ischemic stroke patients. Increased MV or PI in the MCA stem constituted an independent prognostic factor for recurrent vascular events in minor stroke or TIA [19]. Another investigator reported that lower MV and higher PI in the MCA were associated with poor outcomes in ischemic stroke patients, regardless of stroke subtype [20]. In large scale studies, the proximal MCA occlusion or decreased MCA MV on TCD could identify patients with high risk for poor functional outcome in acute ischemic stroke patients with or without recanalization [21–23]. However, unilateral TCD parameters may fail to reveal subtle abnormalities in cerebral hemodynamics. To the best of our knowledge, no study has yet reported the association between TCD parameter asymmetry and prognosis of CS patients.

We found that CS patients with poor outcomes tended to have lower MV and higher PI. This finding is in accordance with other studies [20, 23], but there exists no statistical difference. To overcome the limitation of single TCD parameters of MV or PI, we developed the novel MCA index, which combines the effect of MV and PI. The MCA index was calculated as 100 X (MCA MV + MCA PI X 10) / (MCA MV–MCA PI X 10). We considered that the changes of MV will not be significant because CS patients do not have >50% stenosis in cerebral arteries. Mathematically, the MCA index will become high when MV is low and PI is high. We simulated the MCA index when the MV or the PI values are changed, and we chose the calculation of PI multiplied by 10 to augment differences (described in S1 Table). Multivariable analysis showed that this novel MCA asymmetry index was higher in CS patients with poor outcomes compared to those with good outcomes. Among unilateral parameters, the right proximal MCA index was higher in patients with poor outcomes, whereas the left MCA index was not. However, this may be an incidental finding because the results could differ depending on the study population and lesion location (e.g., the left MCA index may be higher in the left side) (S7 Table). The MCA asymmetry index to augment the differences may be superior because it can reflect hemodynamics and collateral status in bilateral hemispheres.

The overall MCA asymmetry index constituted a strong predictor, and it was two-fold higher in CS patients with poor outcomes compared to those with good outcomes. Moreover, CS patients with an overall MCA asymmetry index >9 were 3.464 times (95% CI, 1.443–8.317) more likely to have poor outcomes at 3 months. In contrast, previously reported predictors, including hemoglobin, ESR, and D-dimer were not associated with short-term functional outcomes [24–26] (described in S2 Table). In acute ischemic stroke, asymmetric TCD parameters exhibited correlations with well-known indicators of neurological deterioration [27, 28], large infarct size [16], and extent of brain edema [29]. Asymmetric MV in the MCA was a marker for underlying carotid stenosis and/or severe stenosis in cerebral arteries [30], and it was also associated with subsequent cerebrovascular events [19]. Moreover, the asymmetric MV was associated with diffusion perfusion mismatch [20], and asymmetric MV was found prior to CT changes [31]. However, previous study was only conducted in asymmetric MV. Because PI is more sensitive to infarct volume and intracranial pressure [16, 18, 23], an asymmetry index which combines both MV and PI might be useful.

We cannot provide the exact mechanism for why the asymmetry index can predict outcomes in CS patients. However, we present the following explanatory hypothesis. First, stroke lesions may disrupt interhemispheric connections, which could result in poor motor recovery [32]. Second, several studies demonstrated that multiple infarct lesions and asymmetry in corticospinal tract activity were independently associated with poor outcomes [33, 34] and decreased executive function [35]. The asymmetrical WMH burden was also associated with higher functional deficit, which was independent of total lesion burden [5]. Thus, symmetric brain function and structure integrity might be critical for optimal functioning and recovery after stroke. Therefore, we assume that asymmetrical TCD parameter can explain poor functional outcomes in CS patients, as brain networks related to functional tasks are bilaterally distributed, and it can be disturbed by not only total lesion burden but also asymmetric hemodynamic derangements [5].

The present study has several limitations. First, our results were derived from routine TCD evaluation. In the study hospital, TCD monitoring was not routinely performed. Because TCD monitoring can simultaneously evaluate TCD parameters in both sides, it possesses some merits to detect asymmetry simultaneously [36]. However, since the time interval of evaluation between both sides was short, the results might be similar. Further comparison studies using TCD monitoring to identify the association between the MCA asymmetry index and outcomes in CS patients might be beneficial. Second, this study was performed using data of a single stroke center and included a population comprised of a single ethnicity. In addition, exclusion of patients who did not undergo TCD or had poor temporal windows on either side may have affected the study results. Finally, comparing affected and unaffected sides is reasonable in specific situations such as anterior circulation lesion, but we failed to present similar results. The difference might be attributable to too small sample size to produce noticeable results (S3 Table). We additionally performed multivariable analysis in 377 CS patients to investigate if the MCA asymmetry index is still an independent predictor after adjusting infarction size. However, the MCA asymmetry index was not independently associated with a poor outcome after adjusting infarction size (S6 Table). It might be related that infarct size was too strong predictor or poor outcome. A previous study showed that the asymmetrical ischemic lesion was related to impaired cerebral vasoreactivity, and then might indicate a poor collateral network in asymmetric infarct lesions [37]. We found that the poor outcome group tended to have more frequent multiple infarctions than good outcome group. Multiple lesions may bring asymmetry in TCD parameters than single small lesions (S4 Table). Therefore, a larger prospective study is needed to confirm our study findings.

## Conclusion

We demonstrated that an asymmetry index using the novel MCA index was associated with short-term functional outcomes in CS patients. Especially, the MCA asymmetry index >9 was a strong independent predictor. Further studies to determine whether the novel MCA asymmetry index can predict long-term outcomes in CS patients are requisite. This novel index should also be tested in other stroke subtypes.

## Supporting information

S1 Table The MCA index according to incremental mean velocity (MV) and pulsatility index (PI). MCA, middle cerebral artery; MV, mean flow velocity; PI, pulsatility index.
(DOCX)

S2 Table Predictors of poor functional outcomes at 3 months. Data were derived from logistic regression analysis; NIHSS, National Institutes of Health Stroke Scale; ESR, erythrocyte sedimentation rate; MCA, middle cerebral artery; OR, odds ratio; CI, confidence interval.
(DOCX)

S3 Table Comparison of baseline characteristics between a good outcome (mRS 0–2) and a poor outcome (mRS 3–6) at 3 months in patients with only anterior circulation lesion. Data are expressed as mean ± SD, median [interquartile rage], or a number (%);
The MCA indices (%) are calculated as 100 X (MCA MV + MCA PI X 10) / (MCA MV–MCA PI X 10); The mean MCA index is calculated as (proximal MCA index + distal MCA index) / 2; The MCA asymmetry index was calculated as 100 X (|Affected MCA index–Unaffected MCA index |) / (Affected MCA index + Unaffected MCA index) / 2; mRS, modified Rankin Scale score; NIHSS, National Institutes of Health Stroke Scale; DWI, Diffusion-weighted magnetic resonance imaging; TCD, transcranial Doppler; Rt, right; Lt, left; MCA, middle cerebral artery; MV, mean flow velocity; PI, pulsatility index.
(DOCX)

S4 Table Demographic characteristics and comparison between a good outcome and a poor outcome in all CS patients. The Multiple infarct lesion is defined as ≥2 infarct origins on DWI; TCD, transcranial Doppler; DWI, Diffusion-weighted magnetic resonance imaging.
(DOCX)

S5 Table Predictors of poor functional outcomes (mRS 2–6) at 3 months in all CS patients. Data were derived from logistic regression analysis; NIHSS, National Institutes of Health Stroke Scale; ESR, erythrocyte sedimentation rate; Rt, right; Lt, left; MCA, middle cerebral artery; OR, odds ratio; CI, confidence interval. * adjusted for sex, age, NIHSS score at admission, hemoglobin, ESR, D-dimer, and Time from admission to TCD.
(DOCX)

S6 Table Predictors of poor functional outcomes at 3 months including infarct volume in all CS patients. Data were derived from logistic regression analysis; NIHSS, National Institutes of Health Stroke Scale; ESR, erythrocyte sedimentation rate; DWI, Diffusion-weighted magnetic resonance imaging; MCA, middle cerebral artery; OR, odds ratio; CI, confidence interval. * adjusted for sex, age, NIHSS score at admission, hemoglobin, ESR, D-dimer, Time from admission to TCD, and DWI infarct volume.
(DOCX)

S7 Table Distribution of infarct lesion between patients with good outcomes (mRS 0–2) and poor outcomes (mRS 3–6) at 3 months. (DOCX)

S1 Fig ROC curve analysis for cutoff value of overall MCA asymmetry index. AUC, area under the curve; MCA, middle cerebral artery.
(DOCX)
