# Supplementary material for: Prediction of functional outcome using the novel asymmetric middle cerebral artery index in cryptogenic stroke patients
Source: PLoS One. 2019 Jan 2;14(1):e0208918. doi: 10.1371/journal.pone.0208918 (PMC6314577; doi:10.1371/journal.pone.0208918)
Supplement: S1 Table — MCA, middle cerebral artery; MV, mean flow velocity; PI, pulsatility index. (DOCX) [file pone.0208918.s001.docx]

**S1 Table. The MCA index according to incremental mean velocity (MV) and pulsatility index (PI)**

| MV (cm/s) | PI | MCA index | Average rate of change |  | MV (cm/s) | PI | MCA_index | Average rate of change |
| --- | --- | --- | --- | --- | --- | --- | --- | --- |
| 50 | 0.7 | 102.840 | 0.050 |  | 50 | 0.71 | 102.881 | 4.122 |
| 51 | 0.7 | 102.783 |  |  | 50 | 0.72 | 102.922 |  |
| 52 | 0.7 | 102.729 |  |  | 50 | 0.73 | 102.963 |  |
| 53 | 0.7 | 102.677 |  |  | 50 | 0.74 | 103.005 |  |
| 54 | 0.7 | 102.627 |  |  | 50 | 0.75 | 103.046 |  |
| 55 | 0.7 | 102.578 |  |  | 50 | 0.76 | 103.087 |  |
| 56 | 0.7 | 102.532 |  |  | 50 | 0.77 | 103.128 |  |
| 57 | 0.7 | 102.487 |  |  | 50 | 0.78 | 103.169 |  |

MCA, middle cerebral artery; MV, mean flow velocity; PI, pulsatility index.
