# Supplementary material for: Prediction of functional outcome using the novel asymmetric middle cerebral artery index in cryptogenic stroke patients
Source: PLoS One. 2019 Jan 2;14(1):e0208918. doi: 10.1371/journal.pone.0208918 (PMC6314577; doi:10.1371/journal.pone.0208918)
Supplement: S2 Table — Data were derived from logistic regression analysis; NIHSS, National Institutes of Health Stroke Scale; ESR, erythrocyte sedimentation rate; MCA, middle cerebral artery; OR, odds ratio; CI, confidence interval. (DOCX) [file pone.0208918.s002.docx]

**S2 Table.** **Predictors of poor functional outcomes at 3 months**

|  | Univariable |  | Multivariable* |  |
| --- | --- | --- | --- | --- |
|  | OR (95% CI) | p-value | OR (95% CI) | p-value |
| Men | 0.540 (0.293 - 0.996) | 0.048 | 0.362 (0.144 - 0.908) | 0.030 |
| Age, y | 1.048 (1.019 - 1.077) | 0.001 | 1.028 (0.988 - 1.071) | 0.175 |
| NIHSS score at admission | 1.285 (1.198 - 1.378) | <0.001 | 1.296 (1.189 - 1.413) | <0.001 |
| Hemoglobin, g/dL | 0.796 (0.696 - 0.910) | 0.001 | 0.939 (0.736 - 1.198) | 0.612 |
| ESR, mm/h | 1.017 (1.005 - 1.029) | 0.007 | 1.015 (0.996 - 1.036) | 0.129 |
| D-dimer, µg/L | 1.000 (1.000 - 1.000) | 0.002 | 1.000 (1.000 - 1.000) | 0.053 |
| Cutoff value of overall MCA asymmetry index >9 | 3.528 (1.897 – 6.561) | <0.001 | 3.464 (1.443 - 8.317) | 0.005 |

Data were derived from logistic regression analysis;

NIHSS, National Institutes of Health Stroke Scale; ESR, erythrocyte sedimentation rate; MCA, middle cerebral artery; OR, odds ratio; CI, confidence interval.
