# Supplementary material for: Prediction of functional outcome using the novel asymmetric middle cerebral artery index in cryptogenic stroke patients
Source: PLoS One. 2019 Jan 2;14(1):e0208918. doi: 10.1371/journal.pone.0208918 (PMC6314577; doi:10.1371/journal.pone.0208918)
Supplement: S3 Table — Data are expressed as mean ± SD, median [interquartile rage], or a number (%); The MCA indices (%) are calculated as 100 X (MCA MV + MCA PI X 10) / (MCA MV–MCA PI X 10); The mean MCA index is calculated as (proximal MCA index + distal MCA index) / 2; The MCA asymmetry index was calculated as 100 X (|Affected MCA index–Unaffected MCA index |) / (Affected MCA index + Unaffected MCA index) / 2; mRS, modified Rankin Scale score; NIHSS, National Institutes of Health Stroke Scale; DWI, Diffusion-weighted magnetic resonance imaging; TCD, transcranial Doppler; Rt, right; Lt, left; MCA, middle cerebral artery; MV, mean flow velocity; PI, pulsatility index. (DOCX) [file pone.0208918.s003.docx]

**S3 Table.** **Comparison of baseline characteristics between a good outcome (mRS 0-2) and a poor outcome (mRS 3-6) at 3 months in patients with only anterior circulation lesion**

|  | Poor outcome  (n=27) | Good outcome  (n=144) | p-value |
| --- | --- | --- | --- |
| Age, y | 56.59 ± 12.31 | 61.67 ± 12.06 | 0.124 |
| Men | 15 (55.6) | 100 (69.4) | 0.158 |
| NIHSS score at admission | 7.5 [4.0, 12.3] | 2.0 [1.0, 4.0] | <0.001 |
| Time from admission to TCD (day) | 5.23 ± 3.40 | 3.36 ± 2.96 | 0.007 |
| Systolic blood pressure (mmHg) | 155.6 ± 22.0 | 159.9 ± 28.1 | 0.484 |
| Diastolic blood pressure (mmHg) | 85.9 ± 10.6 | 88.7 ± 15.6 | 0.382 |
| DWI infarct volume, mL | 3.60 [1.52, 13.52] | 1.46 [0.41, 4.39] | 0.002 |
| Thrombolysis therapy | 2 (7.4) | 11 (7.6) | 0.967 |
|  |  |  |  |
| **Vessel status** |  |  |  |
| Prox LMCA stenosis or occlusion | 1 (25.0) | 3 (23.1) | 1.000 |
| Prox RMCA stenosis or occlusion | 1 (25.0) | 2 (12.5) | 0.509 |
| Dist LMCA stenosis or occlusion | 2 (66.7) | 12 (75.0) | 1.000 |
| Dist RMCA stenosis or occlusion | 0 (0.0) | 5 (55.6) | NA |
| LICA stenosis or occlusion | 0 (0.0) | 2 (7.7) | 1.000 |
| RICA stenosis or occlusion | 0 (0.0) | 3 (15.0) | 1.000 |
|  |  |  |  |
| **TCD parameters** |  |  |  |
| Affected proximal MCA MV | 61.5 [ 42.0, 79.5] | 57.0 [ 47.0, 71.0] | 0.757 |
| Affected proximal MCA PI  Affected distal MCA MV  Affected distal MCA PI  Unaffected proximal MCA MV  Unaffected proximal MCA PI  Unaffected proximal MCA MV  Unaffected proximal MCA PI | 0.91 [0.76, 1.03]  61.0 [ 49.3, 75.3]  0.92 [0.73, 1.10]  56.0 [50.0, 77.3]  0.89 [0.71, 0.96]  60.5 [46.5, 66.25]  0.87 [0.76, 1.10] | 0.85 [0.73, 0.94]  57.0 [ 46.5, 71.0]  0.83 [0.72, 0.95]  59.0 [47.5, 72.5]  0.83 [0.76, 1.00]  57.0 [46.0, 70.5]  0.82 [0.75, 1.00] | 0.191  0.327  0.084  0.679  0.954  0.879  0.276 |
| **Novel TCD parameters (%)** |  |  |  |
| Affected proximal MCA index | 134.68 [ 124.04, 161.93] | 135.29 [124.32, 147.52] | 0.820 |
| Unaffected proximal MCA index | 137.74 [ 123.93, 143.91] | 132.97 [ 124.38, 145.80] | 0.855 |
| Proximal MCA asymmetry index | 9.06 [2.35, 16.91] | 5.84 [2.85, 10.55] | 0.288 |
|  |  |  |  |
| Affected distal MCA index | 136.21 [ 126.77, 146.37] | 133.82 [ 124.79, 146.91] | 0.800 |
| Unaffected distal MCA index | 135.47 [ 127.93, 143.79] | 134.31 [ 123.92, 146.19] | 0.609 |
| Distal MCA asymmetry index | 8.18 [2.51, 15.41] | 4.92 [2.92, 11.27] | 0.421 |
|  |  |  |  |
| Affected mean MCA index | 134.83 [ 122.77, 152.89] | 134.61 [ 124.50, 146.38] | 0.830 |
| Unaffected mean MCA index | 135.52 [ 128.46, 141.29] | 132.74 [ 125.43, 146.48] | 0.629 |
| Overall MCA asymmetry index | 7.68 [2.81, 13.77] | 5.57 [2.86, 9.29] | 0.354 |

Data are expressed as mean ± SD, median [interquartile rage], or a number (%);

The MCA indexes (%) are calculated as 100 X (MCA MV + MCA PI X 10) / (MCA MV – MCA PI X 10); The mean MCA index is calculated as (proximal MCA index + distal MCA index) / 2;

The MCA asymmetry index was calculated as 100 X (|Affected MCA index – Unaffected MCA index |) / (Affected MCA index + Unaffected MCA index) / 2;

mRS, modified Rankin Scale score; NIHSS, National Institutes of Health Stroke Scale; DWI, Diffusion-weighted magnetic resonance imaging; TCD, transcranial Doppler; Rt, right; Lt, left; MCA, middle cerebral artery; MV, mean flow velocity; PI, pulsatility index.
