# Supplementary material for: Prediction of functional outcome using the novel asymmetric middle cerebral artery index in cryptogenic stroke patients
Source: PLoS One. 2019 Jan 2;14(1):e0208918. doi: 10.1371/journal.pone.0208918 (PMC6314577; doi:10.1371/journal.pone.0208918)
Supplement: S4 Table — The Multiple infarct lesion is defined as ≥2 infarct origins on DWI; TCD, transcranial Doppler; DWI, Diffusion-weighted magnetic resonance imaging. (DOCX) [file pone.0208918.s004.docx]

**S4 Table. Demographic characteristics and comparison between a good outcome and a poor outcome in all CS patients**

|  | Total | Good outcome  (mRS 0-2; n=325) | Poor outcome  (mRS 3-6; n=52) | p-value |
| --- | --- | --- | --- | --- |
|  | (n=377) |  |  |  |
| Time from admission to TCD (day) | 3.0 [2.0, 4.0] | 3.0 [2.0, 4.0] | 4.0 [3.0, 6.0] | <0.001 |
| Systolic blood pressure (mmHg) | 155.0 [136.0, 177.0] | 156.0 [137.0, 179.0] | 153.0 [132.0, 169.0] | 0.109 |
| Diastolic blood pressure (mmHg) | 87.0 [77.0, 96.0] | 87.0 [78.0, 97.0] | 85.0 [75.0, 90.0] | 0.060 |
| DWI infarct volume, mL | 1.71 [0.44, 4.88] | 1.33 [0.38, 19.50] | 3.76 [1.56, 28.78] | 0.002 |
| Multiple infarct lesion | 73 (19.4 ) | 59 (18.2) | 14 (26.9) | 0.137 |

The Multiple infarct lesion is defined as ≥2 infarct origins on DWI; TCD, transcranial Doppler; DWI, Diffusion-weighted magnetic resonance imaging.
