# Supplementary material for: Prediction of functional outcome using the novel asymmetric middle cerebral artery index in cryptogenic stroke patients
Source: PLoS One. 2019 Jan 2;14(1):e0208918. doi: 10.1371/journal.pone.0208918 (PMC6314577; doi:10.1371/journal.pone.0208918)
Supplement: S5 Table — Data were derived from logistic regression analysis; NIHSS, National Institutes of Health Stroke Scale; ESR, erythrocyte sedimentation rate; Rt, right; Lt, left; MCA, middle cerebral artery; OR, odds ratio; CI, confidence interval. * adjusted for sex, age, NIHSS score at admission, hemoglobin, ESR, D-dimer, and Time from admission to TCD. (DOCX) [file pone.0208918.s005.docx]

**S5 Table. Predictors of poor functional outcomes (mRS 2-6) at 3 months in all CS patients**

|  | Univariable |  | Multivariable* |  |
| --- | --- | --- | --- | --- |
|  | OR (95% CI) | p-value | OR (95% CI) | p-value |
| Men | 0.647 (0.393 – 1.066) | 0.088 |  |  |
| Age, y | 1.028 (1.007 - 1.049) | 0.007 |  |  |
| NIHSS score at admission | 1.393 (1.280 - 1.517) | <0.001 |  |  |
| Hemoglobin, g/dL | 0.866 (0.774 - 0.969) | 0.012 |  |  |
| ESR, mm/h | 1.009 (0.999 - 1.020) | 0.083 |  |  |
| D-dimer, µg/L | 1.000 (1.000 - 1.000) | 0.020 |  |  |
| Time from admission to TCD (day) | 1.143 (1.041 – 1.255) | 0.005 |  |  |
| **Novel TCD parameters** |  |  |  |  |
| Rt proximal MCA index | 1.007 (0.999 - 1.015) | 0.068 | 1.013 (1.003 - 1.023) | 0.013 |
| Lt proximal MCA index | 1.009 (1.000 - 1.018) | 0.039 | 1.013 (1.002 - 1.025) | 0.024 |
| Proximal MCA asymmetry index | 1.028 (1.001 - 1.055) | 0.038 | 1.023 (0.991 - 1.055) | 0.162 |
| Rt distal MCA index | 1.005 (0.999 - 1.010) | 0.084 | 1.004 (0.998 - 1.011) | 0.172 |
| Lt distal MCA index | 1.008 (1.000 - 1.015) | 0.041 | 1.009 (0.999 - 1.018) | 0.065 |
| Distal MCA asymmetry index | 1.025 (1.008 - 1.041) | 0.003 | 1.019 (1.000 - 1.038) | 0.051 |
| Rt mean MCA index | 1.007 (0.999 - 1.015) | 0.071 | 1.009 (0.998 - 1.019) | 0.096 |
| Lt mean MCA index | 1.013 (1.003 - 1.022) | 0.009 | 1.016 (1.003 - 1.028) | 0.014 |
| Overall MCA asymmetry index | 1.056 (1.028 - 1.085) | <0.001 | 1.053 (1.018 - 1.090) | 0.003 |
| Cutoff value of overall MCA asymmetry index >9 | 2.996 (1.834 – 4.896) | <0.001 | 3.351 (1.680 – 6.685) | 0.001 |

Data were derived from logistic regression analysis;

NIHSS, National Institutes of Health Stroke Scale; ESR, erythrocyte sedimentation rate; Rt, right; Lt, left; MCA, middle cerebral artery; OR, odds ratio; CI, confidence interval.

* adjusted for sex, age, NIHSS score at admission, hemoglobin, ESR, D-dimer, and Time from admission to TCD.

**S6 Table. Predictors of poor functional outcomes at 3 months including infarct volume in all CS patients**

|  | Univariable |  | Multivariable* |  |
| --- | --- | --- | --- | --- |
|  | OR (95% CI) | p-value | OR (95% CI) | p-value |
| Men | 0.647 (0.393 - 1.066) | 0.088 |  |  |
| Age, y | 1.028 (1.007 - 1.049) | 0.007 |  |  |
| NIHSS score at admission | 1.393 (1.280 - 1.517) | <0.001 |  |  |
| Hemoglobin, g/dL | 0.866 (0.774 - 0.969) | 0.012 |  |  |
| ESR, mm/h | 1.009 (0.999 - 1.020) | 0.083 |  |  |
| D-dimer, µg/L | 1.000 (1.000 - 1.000) | 0.020 |  |  |
| Time from admission to TCD (day) | 1.143 (1.041 - 1.255) | 0.005 |  |  |
| DWI infarct volume, mL | 1.024 (1.008 - 1.040) | 0.004 |  |  |
| **Novel TCD parameters** |  |  |  |  |
| Proximal MCA asymmetry index | 1.038 (1.007 - 1.069) | 0.014 | 1.039 (0.981 - 1.100) | 0.194 |
| Distal MCA asymmetry index | 1.027 (1.009 - 1.044) | 0.002 | 1.000 (0.970 - 1.031) | 0.999 |
| Overall MCA asymmetry index | 1.055 (1.026 - 1.086) | <0.001 | 1.022 (0.966 - 1.081) | 0.445 |
| Cutoff value of overall MCA asymmetry index >9 | 3.528 (1.897 - 6.561) | <0.001 | 2.169 (0.584 - 8.054) | 0.247 |

Data were derived from logistic regression analysis;

NIHSS, National Institutes of Health Stroke Scale; ESR, erythrocyte sedimentation rate; DWI, Diffusion-weighted magnetic resonance imaging; MCA, middle cerebral artery; OR, odds ratio; CI, confidence interval.

* adjusted for sex, age, NIHSS score at admission, hemoglobin, ESR, D-dimer, Time from admission to TCD, and DWI infarct volume.

**S7 Table.** **Distribution of infarct lesion between patients with good outcomes (mRS 0-2) and poor outcomes (mRS 3-6) at 3 months**

|  | Good outcome  (n=325) | Poor outcome  (n=52) | p-value |
| --- | --- | --- | --- |
| Left side | 147 (45.2) | 20 (38.5) | 0.407 |
| Right side | 99 (30.5) | 17 (32.7) |  |
| Central | 1 (0.3) | 1 (1.9) |  |
| Bilateral | 52 (17.5) | 12 (23.1) |  |


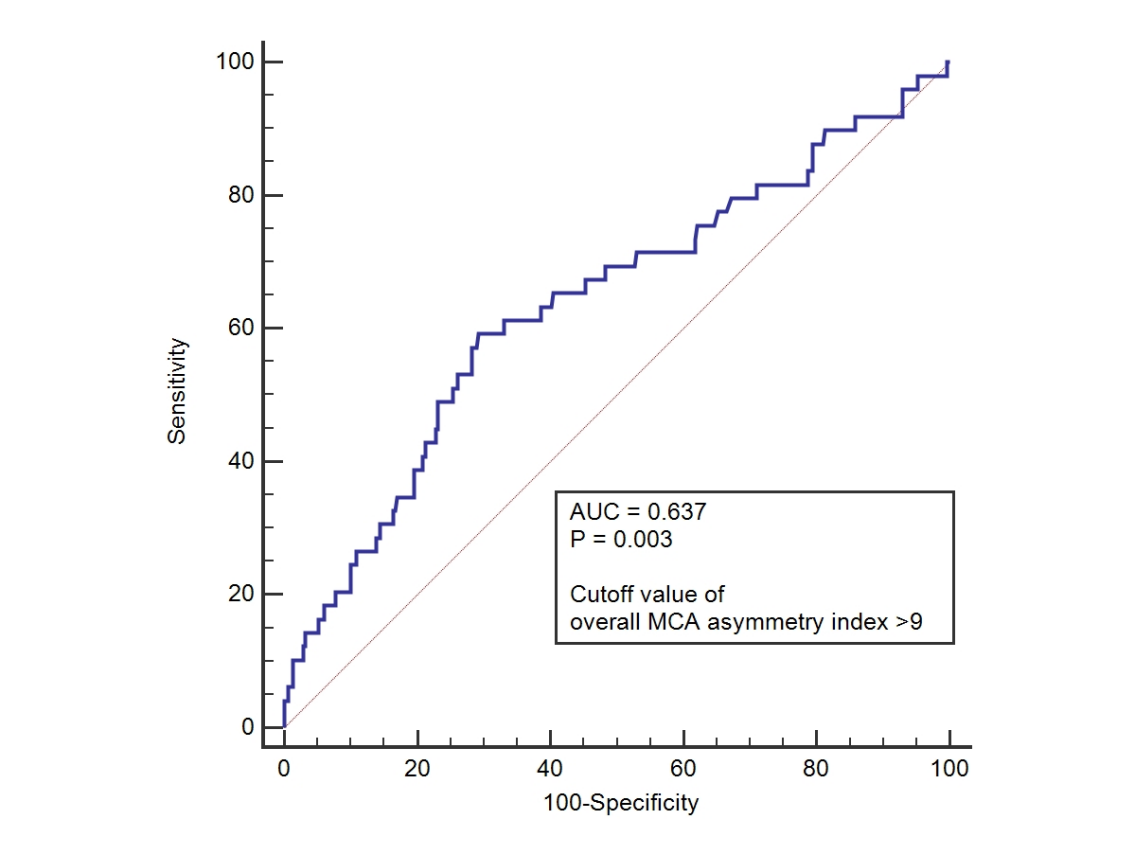


**S1 Figure.** **ROC curve analysis for cutoff value of overall MCA asymmetry index**

AUC, area under the curve; MCA, middle cerebral artery.
