# Supplementary material for: Prediction of functional outcome using the novel asymmetric middle cerebral artery index in cryptogenic stroke patients
Source: PLoS One. 2019 Jan 2;14(1):e0208918. doi: 10.1371/journal.pone.0208918 (PMC6314577; doi:10.1371/journal.pone.0208918)
Supplement: S6 Table — Data were derived from logistic regression analysis; NIHSS, National Institutes of Health Stroke Scale; ESR, erythrocyte sedimentation rate; DWI, Diffusion-weighted magnetic resonance imaging; MCA, middle cerebral artery; OR, odds ratio; CI, confidence interval. * adjusted for sex, age, NIHSS score at admission, hemoglobin, ESR, D-dimer, Time from admission to TCD, and DWI infarct volume. (DOCX) [file pone.0208918.s006.docx]

**S6 Table. Predictors of poor functional outcomes at 3 months including infarct volume in all CS patients**

|  | Univariable |  | Multivariable* |  |
| --- | --- | --- | --- | --- |
|  | OR (95% CI) | p-value | OR (95% CI) | p-value |
| Men | 0.647 (0.393 - 1.066) | 0.088 |  |  |
| Age, y | 1.028 (1.007 - 1.049) | 0.007 |  |  |
| NIHSS score at admission | 1.393 (1.280 - 1.517) | <0.001 |  |  |
| Hemoglobin, g/dL | 0.866 (0.774 - 0.969) | 0.012 |  |  |
| ESR, mm/h | 1.009 (0.999 - 1.020) | 0.083 |  |  |
| D-dimer, µg/L | 1.000 (1.000 - 1.000) | 0.020 |  |  |
| Time from admission to TCD (day) | 1.143 (1.041 - 1.255) | 0.005 |  |  |
| DWI infarct volume, mL | 1.024 (1.008 - 1.040) | 0.004 |  |  |
| **Novel TCD parameters** |  |  |  |  |
| Proximal MCA asymmetry index | 1.038 (1.007 - 1.069) | 0.014 | 1.039 (0.981 - 1.100) | 0.194 |
| Distal MCA asymmetry index | 1.027 (1.009 - 1.044) | 0.002 | 1.000 (0.970 - 1.031) | 0.999 |
| Overall MCA asymmetry index | 1.055 (1.026 - 1.086) | <0.001 | 1.022 (0.966 - 1.081) | 0.445 |
| Cutoff value of overall MCA asymmetry index >9 | 3.528 (1.897 - 6.561) | <0.001 | 2.169 (0.584 - 8.054) | 0.247 |

Data were derived from logistic regression analysis;

NIHSS, National Institutes of Health Stroke Scale; ESR, erythrocyte sedimentation rate; DWI, Diffusion-weighted magnetic resonance imaging; MCA, middle cerebral artery; OR, odds ratio; CI, confidence interval.

* adjusted for sex, age, NIHSS score at admission, hemoglobin, ESR, D-dimer, Time from admission to TCD, and DWI infarct volume.
