# Supplementary material for: Prediction of functional outcome using the novel asymmetric middle cerebral artery index in cryptogenic stroke patients
Source: PLoS One. 2019 Jan 2;14(1):e0208918. doi: 10.1371/journal.pone.0208918 (PMC6314577; doi:10.1371/journal.pone.0208918)
Supplement: S7 Table — (DOCX) [file pone.0208918.s007.docx]

**S7 Table.** **Distribution of infarct lesion between patients with good outcomes (mRS 0-2) and poor outcomes (mRS 3-6) at 3 months**

|  | Good outcome  (n=325) | Poor outcome  (n=52) | p-value |
| --- | --- | --- | --- |
| Left side | 147 (45.2) | 20 (38.5) | 0.407 |
| Right side | 99 (30.5) | 17 (32.7) |  |
| Central | 1 (0.3) | 1 (1.9) |  |
| Bilateral | 52 (17.5) | 12 (23.1) |  |
