# Supplementary material for: Prediction of functional outcome using the novel asymmetric middle cerebral artery index in cryptogenic stroke patients
Source: PLoS One. 2019 Jan 2;14(1):e0208918. doi: 10.1371/journal.pone.0208918 (PMC6314577; doi:10.1371/journal.pone.0208918)
Supplement: S1 Fig — AUC, area under the curve; MCA, middle cerebral artery. (DOCX) [file pone.0208918.s008.docx]

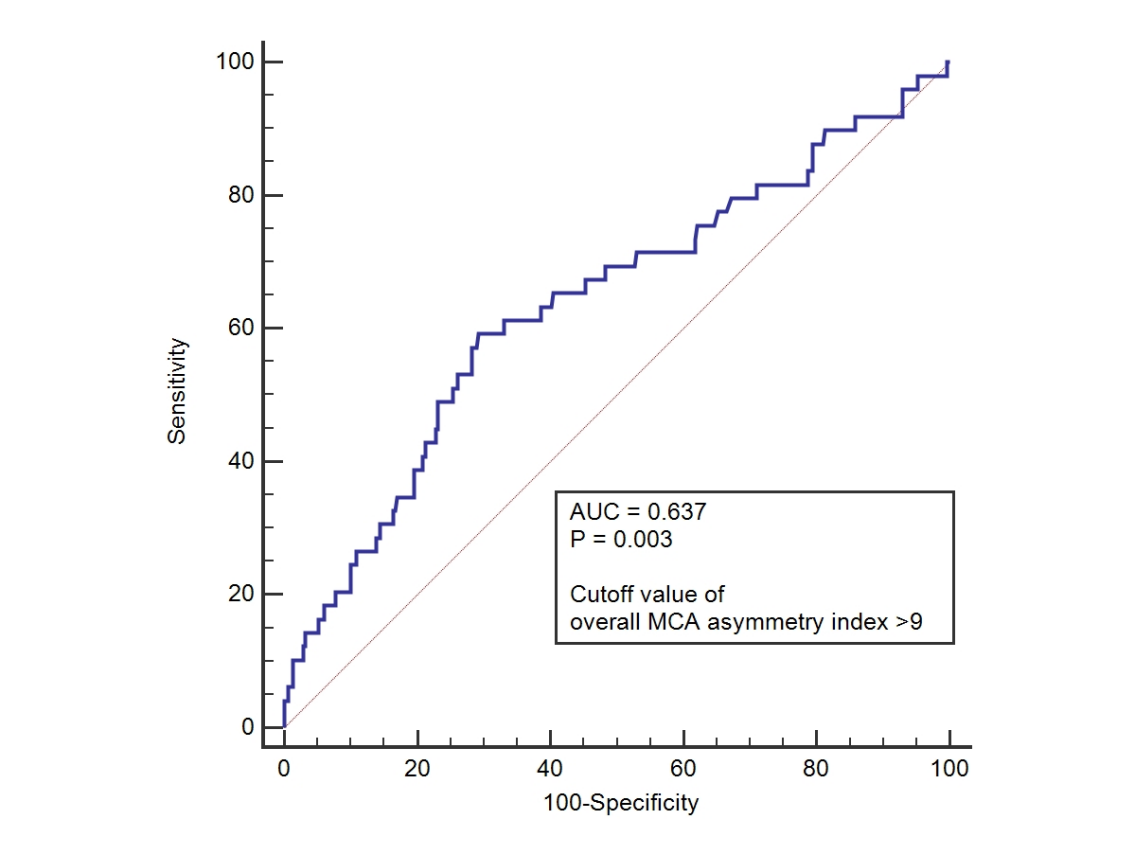


**S1 Fig.** **ROC curve analysis for cutoff value of overall MCA asymmetry index.**

AUC, area under the curve; MCA, middle cerebral artery.
